# Phylogenetic analysis of cryptic speciation in the polychaete *Pygospio elegans*

**DOI:** 10.1002/ece3.226

**Published:** 2012-05

**Authors:** J E Kesäniemi, P D Rawson, S M Lindsay, K E Knott

**Affiliations:** 1 Department of Biological and Environmental Science, University of JyväskyläP.O. Box 35, FI-40014, Finland; 2 School of Marine Sciences, University of MaineOrono, Maine 04469-5751

**Keywords:** COI, developmental mode, larvae, population structure

## Abstract

Development in marine invertebrate species can take place through a variety of modes and larval forms, but within a species, developmental mode is typically uniform. Poecilogony refers to the presence of more than one mode of development within a single species. True poecilogony is rare, however, and in some cases, apparent poecilogony is actually the result of variation in development mode among recently diverged cryptic species. We used a phylogenetic approach to examine whether poecilogony in the marine polychaete worm, *Pygospio elegans*, is the result of cryptic speciation. Populations of worms identified as *P. elegansooded*, and intermediate larvae; these modes are found both within and among populations. We examined sequence variation among partial mitochondrial cytochrome c oxidase subunit I sequences obtained for 279 individual worms sampled across broad geographic and environmental scales. Despite a large number of unique haplotypes (121 haplotypes from 279 individuals), sequence divergence among European samples was low (1.7%) with most of the sequence variation observed within populations, relative to the variation among regions. More importantly, we observed common haplotypes that were widespread among the populations we sampled, and the two most common haplotypes were shared between populations differing in developmental mode. Thus, our results support an earlier conclusion of poecilogony in *P elegans*. In addition, predominantly planktonic populations had a larger number of population-specific low-frequency haplotypes. This finding is largely consistent with interspecies comparisons showing high diversity for species with planktonic developmental modes in contrast to low diversity in species with brooded developmental modes.

## Introduction

Most marine invertebrates have complex life cycles and show a diverse range of larval developmental modes. Developmental mode is often defined as discrete categories describing characteristics of larvae, or larval types (Levin and Bridges 1995). For example, larvae can be planktonic (pelagic) or benthic, feeding or nonfeeding, brooded or free-living, and a combination of multiple descriptors is often necessary for a complete definition of developmental mode (e.g., McEdward and Janies 1993; Collin 2003; Raff and Byrne 2006). Developmental mode is an important aspect of invertebrate life histories, with wide-ranging consequences affecting, for example, development time, mortality, and dispersal potential (Levin and Bridges 1995). Understanding the consequences and evolution of different developmental modes is, on one hand, aided by our tendency to categorize it as discrete types. On the other hand, such definitions may also lead us to overlook intermediate or facultatively varying forms that do not fit definitions of discrete developmental modes (Allen and Pernet 2007).

Many different developmental modes may be observed within genera or larger taxonomic groups, but typically only one developmental mode exists within a single species. In rare cases, species may express two or more development modes. The term poecilogony (Giard 1905 cited in Krug 2009) has been used to describe such developmental mode polymorphism. In poecilogonous species, multiple developmental modes are observed, either within or among different populations of a single species. True poecilogony has been documented within spionid worms (e.g., *Streblospio benedicti*, Levin 1984, and *Boccardia proboscidia*, Gibson 1997; Oyarzun et al. 2011) and in sacoglossan sea slugs (reviewed in Krug 2007, 2009). However, in a number of cases, what were originally described as poecilogonous species have turned out to be morphologically cryptic species with species-specific developmental modes (see Hoagland and Robertson 1988). The rarity of true poecilogony has led some authors to suggest that there are costs associated with polymorphic development and that poecilogony is a transient stage of speciation co-occurring with developmental mode transitions (Gibson and Gibson 2004; Ellingson and Krug 2006). Alternatively, poecilogony might be an advantageous plastic response, and a potential bet-hedging strategy, to enhance offspring success in the face of changing environmental conditions (Krug 2007).

**Figure 1:**
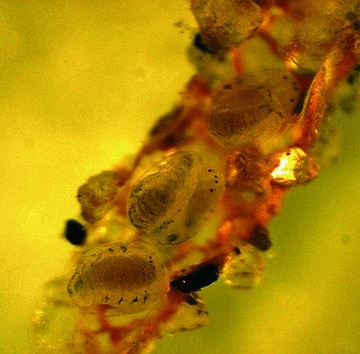
Brooded *Pygospio elegans* larvae in capsules (from Ängsö, Finland). The capsules (approx. 0.5 mm long, each containing one to two larvae) are visible after breaking down the sand tube. Photo credit: Jenni Kesäniemi.

One possible poecilogonous species is *Pygospio elegans* Claparède, a small, sedentary, tube-building spionid polychaete worm, widely distributed in the northern hemisphere (Muus 1967; Anger 1984). After internal fertilization (Hannerz 1956), females deposit embryos and yolky nurse eggs in capsules inside the maternal tube. Different larvae emerge from the capsules depending on the relative number of embryos and nurse eggs laid by the mother; there are no initial differences in embryo size (Söderström 1920; Hannerz 1956; Rasmussen 1973; Anger et al. 1986; Blake and Arnofsky 1999, pers. obs.). Here, we define planktonic larvae as those that emerge when they are 3-setigers long (typically >20 embryos laid per capsule with few or no nurse eggs). The larvae develop long swimming setae and actively swim and feed in the water column (Hannerz 1956). Brooded larvae, on the other hand, do not have swimming setae and remain inside the capsules for a longer period subsisting only on nurse eggs (typically one to two embryos laid per capsule, Fig. 1). These larvae lack a pelagic phase during development and metamorphose into juveniles soon after their emergence from the capsules at 14–20 setigers. An intermediate type of larva also occurs (4–10 embryos laid per capsule; Hannerz 1956, pers. obs.). After emergence at approximately 10 setigers, these larvae have a short pelagic phase. Despite their differences, all larval types metamorphose into morphologically and ecologically identical adults.

Monitoring reproduction in *P. elegans* is laborious and has been done exhaustively in only a few populations. There have been some observations of different larval forms simultaneously within a single population (Rasmussen 1973; Gudmundsson 1985, pers. obs.), providing some evidence that *P. elegans* is a true poecilogonous species. However, whether or not a single individual can produce multiple larval types is not clear (but, see Fig. 30 in Rasmussen 1973). Hannerz (1956) and Rasmussen (1973) hypothesized that developmental mode polymorphism in *P. elegans* is in fact variation within a single developmental mode, reflecting plastic responses to environmental variation. This hypothesis was based on observations that in some populations different larvae are produced seasonally. However, neither simultaneous nor seasonal production of different larvae in a single population is universal. More commonly, among population differences in developmental mode are noted, and some populations have even been considered “fixed” for a particular developmental mode since no other modes have been observed during repeated sampling from these populations (Anger 1984; Morgan et al. 1999; Bolam 2004, pers. obs.). The presence of “fixed” populations differing in developmental mode raises suspicion that cryptic species may be present. This suspicion was strengthened when Anger (1984) found that experimental exposure of worms from several “fixed” populations to different salinities and temperatures did not induce a change in developmental mode. No correlations between other environmental variables and developmental mode have been noted in the literature, but few experimental tests have been performed. Changes in density and food supply did not induce changes in developmental mode in *P. elegans* collected from Somme Bay, France (Morgan 1997), but in North America, low density has apparently increased the frequency of asexual reproduction in *P. elegans* (Wilson 1983).

To clarify the species status of *P. elegans* populations, Morgan and colleagues (1999) examined population structure among four potentially “fixed” populations in the English Channel differing in developmental mode. They found high genetic similarity and potentially high gene flow among the *P. elegans* populations, and concluded that the species is poecilogonous. Nevertheless, due to the limited scope of their study and the rarity of poecilogony, the question of poecilogony versus cryptic speciation still remains. We addressed this question by surveying variation in a portion of the mitochondrial gene cytochrome c oxidase subunit I using haplotype network and phylogenetic methods, and using a DNA sequence-based criterion advocated in DNA barcoding studies to assess the presence of cryptic species. Our samples covered both a broad geographical area and a range of environmental conditions. For some populations, there were also data available regarding the predominant developmental mode among individuals. The large dataset also allowed us to investigate within-population diversity in our study populations. We hypothesized that *P. elegans* is indeed a poecilogonous species, despite apparent divergence of populations in developmental mode.

## Materials and Methods

### Sample collection and molecular methods

**Figure 2:**
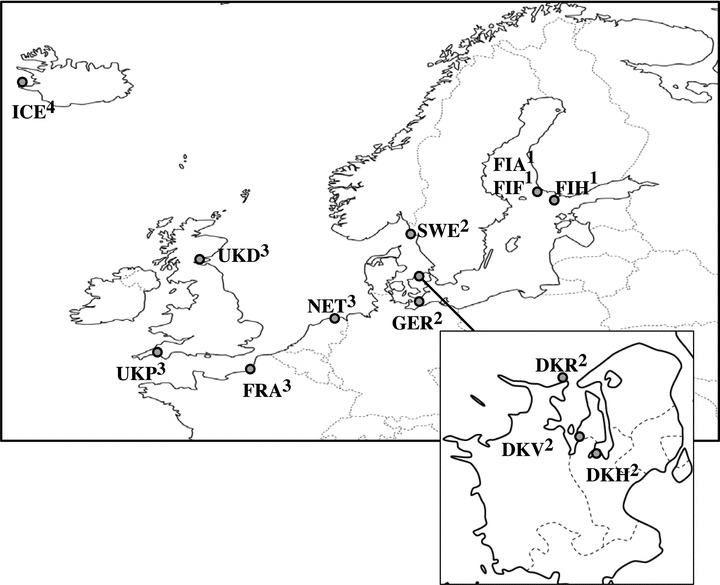
European sampling sites labeled according to their abbreviations in Table 1. Sites FIA (Ängsö) and FIF (Fårö) are located in the Finnish archipelago, approximately 20 km apart. Regional grouping of populations for the hierarchical AMOVA analysis are indicated with numbered superscripts: 1. Northern Baltic Sea: Finland, 2. Southern Baltic Sea: Denmark, Germany, Sweden, 3. North Sea + Wadden Sea + English Channel: UK, France, the Netherlands, and 4. North Atlantic Ocean: Iceland.

Adult *P. elegans* were collected between 2007 and 2010 from 14 locations in Europe (Fig. 2) and three locations in the United States (east coast: Maine and west coast: Washington). In Europe, populations from the Baltic Sea (Finland, Germany, Denmark, Sweden), Wadden Sea (the Netherlands, Schiermonnikoog Island), North Sea (Edinburgh, UK), the English Channel (Plymouth, UK, and Somme Bay, France), White Sea (Russia), and the North Atlantic Ocean (Iceland) were sampled (Fig. 2, Table 1). Several colleagues enabled the collecting effort (see Acknowledgements). At most locations, the samples were collected from the shallow intertidal zone (0.1–1 m). The two samples from the Finnish archipelago (Ängsö and Fårö) were collected by scuba from 2–5 m deep water. Samples from Germany were collected from 18-m depth.

**Table 1:**
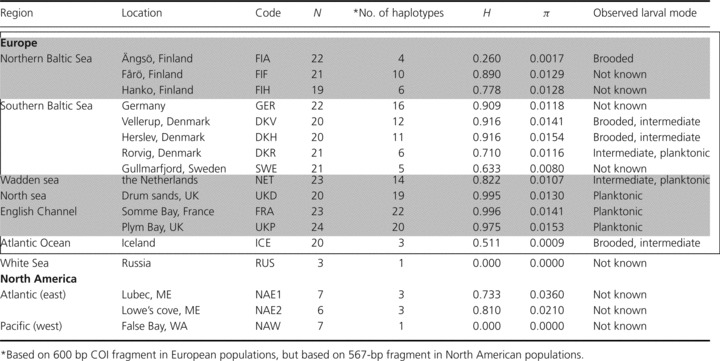
Sampling location information, population codes, diversity measurements, and observed larval modes of the populations. *N*= number of individuals in the genetic analysis, *H*= haplotype diversity, and π= nucleotide diversity. Box indicates the European populations with sufficient sample size used in diversity and demographic analyses as well as hierarchical analyses of population structure (AMOVA). Groups (regions) defined for AMOVA analysis are shaded.

At the time of collecting, the adult worms and sand tubes were examined for signs of larvae or egg capsules and then preserved in ethanol (94–99%). Using these observations, and information from previous studies of *P. elegans*’ reproduction and development (i.e., Rasmussen 1973; Morgan et al. 1999; Bolam 2004), we characterized the sampling locations by the different larval developmental modes observed (Table 1). This characterization is tentative, since we were unable to survey all populations exhaustively, but represents our best knowledge of the predominant developmental mode in the populations. Additional sampling at the same sites has confirmed our characterization of developmental mode (pers. obs.) but at some sites we have not observed any signs of sexual reproduction and so a predominant developmental mode is not known.

From the European samples, genomic DNA was extracted using the DNeasy Blood and Tissue extraction kit (Qiagen, Germany) and a KingFisher magnetic processor (ThermoScientific, MA, USA). A 600-bp fragment of the cytochrome c oxidase subunit I (COI) gene was amplified using species-specific primers (PeCox1 F 5′– TAT AGG CCT TTG ATC AGG AAC – 3′, PeCox1 R 5′– AGG GTC TCC GCC TCC TGT – 3′). Polymerase chain reactions (PCRs) were performed in 20 µL reactions containing 1 µL of the DNA extract, 3 mM MgCl_2_ (Biotools, Spain), 200 µM of each dNTP (Fermentas, Germany), 0.5 µM of each primer (TAG Copenhagen, Denmark), 0.1 U of Taq polymerase, and 1 X of PCR Buffer (Biotools). Reaction conditions included an initial de- naturation step at 94°C for 2 min, then 35 cycles of denaturation at 94°C for 15 s, annealing at 55°C for 15 s, and extension at 72°C for 45 s, followed by a final extension at 72°C for 2 min. For sequencing, the PCR products were treated with Exonuclease I and Shrimp alkaline phosphatase (Fermentas), cycle sequenced in both directions using the BigDye v.3.1 kit, and visualized with an ABI 3130xl Genetic Analyzer and Sequencing Analysis v.5.2. software (all Applied Biosystems, CA, USA).

DNA extraction, amplification, and sequencing of the North American samples followed similar protocols, but sequencing artifacts at the 5′ end of the resulting sequences reduced the length of high-quality sequence reads for these samples. To be conservative, we analyzed a shorter fragment of the COI gene (567 bp) when North American samples were included. In analyses involving only the European samples, the 600-bp fragment was used.

### Haplotype network and phylogenetic analyses

Sequences were aligned using the ClustalW option of MEGA 4 (Tamura et al. 2007). For these analyses, the 567-bp fragment of the COI gene was used and all individuals were included. To examine the relationship between the haplotypes, a minimum spanning network was constructed with Arlequin v.3.5.1.2. (Excoffier and Lischer 2010) and visualized with HapStar (Teacher and Griffiths 2010).

For phylogenetic analyses, a single representative of each haplotype was used. JModeltest (Posada 2008) was used to find the optimal model of sequence evolution for the COI data (selected using the Akaike information criterion, AIC). The general time reversible model with invariant positions and gamma-distributed rates (GTR + I + G) was selected and used in tree reconstruction. Sequence divergence was estimated with MEGA 4 using a gamma shape parameter of 0.637 (according to JModeltest) and the Tamura Nei substitution model since the GTR model is not available in MEGA 4.

For tree reconstruction, we explored both maximum likelihood and Bayesian analyses. Bayesian analysis was conducted with MrBayes v.3.1.2. (Ronquist and Huelsenbeck 2003). MCMC (Markov Chain Monte Carlo) chains (one cold and three heated chains) were run for 4 million generations, trees were sampled every 100 generations, and 25% of the trees were discarded in the burnin. All parameters were estimated in the analysis. Posterior probabilities were used to assess clade support, with 80% used as the minimum cutoff. Maximum likelihood analysis was conducted with PhyML 3.0. (Guindon and Gascuel 2003). All parameters were estimated in the analysis except the gamma shape parameter, which was set to 0.637 according to the results from JModeltest. Bootstrap analysis with 1000 replicates provided an estimate of clade support, with 70% used as the minimum cutoff. After analysis, trees were rooted along the lineage leading to most of the North American haplotypes (also the longest branch). Trees were visualized using FigTree v.1.2.2. (http://tree.bio.ed.ac.uk/software/figtree/).

### Analysis of genetic diversity

Our genetic diversity analyses focused on populations with sufficient sample sizes for making robust estimates, so the Russian sample (*n*= 3) and the North American samples (*n*= 6–7) were excluded. In these analyses, the 600-bp fragment of the COI gene was used. Haplotype diversity and nucleotide diversity for each population were calculated with Arlequin v.3.5.1.2. (Excoffier and Lischer 2010), which was also used to estimate population structure (Φ_ST_) via a hierarchical analysis of molecular variance (AMOVA). In the AMOVA analysis, sequences were grouped according to geographical regions (four groups: Northern Baltic Sea; Southern Baltic Sea; North Sea + Wadden Sea + English Channel; and North Atlantic Ocean; 10,000 permutations). Population structure was also investigated using BAPS 5.3 (Corander and Tang 2007), a Bayesian model-based clustering method that can use sequence data. In these analyses, the maximum number of clusters (*K*) was set from two to 13, and for each the analysis was run 10 times. In the end, the *K* with the highest likelihood was chosen to describe the samples.

Exploratory analyses tested whether differences in haplotype and nucleotide diversity measures were evident among the European populations with different developmental mode. Here, planktonic populations (UKP, UKD, FRA, see Table 1) were compared to populations that produce brooded or intermediate type larvae (FIA, DKV, DKH, DKR, NET, ICE). This comparison is contingent on our definition of predominant developmental mode (see Table 1), so populations where developmental mode is not known (FIF, FIH, GER, SWE, and RUS) were excluded. For these comparisons, Mann–Whitney *U* tests were performed using PASW Statistics 18 (SPSS, Inc., 2009, Chicago, IL, http://www.spss.com).

To assess if European populations (excluding RUS) have gone through a recent population expansion, Fu's *F*s neutrality test was calculated. Fu's test (which is based on the haplotype distribution; Fu 1997) was used because it is thought to be better at revealing signs of population expansion than Tajima's *D* test (Fu 1997; Schneider and Excoffier 1999). Tajima's *D* (Tajima 1989) and Fu and Li's *F* (Fu and Li 1993) were also calculated to test for neutrality of the sequences. Mismatch distributions, the frequencies of observed pairwise differences between haplotypes, were calculated for each European population. Also *R*_2_ (Ramos-Onsins and Rozas 2002) and raggedness statistics (rg, Harpending 1994) with confidence intervals based on coalescent simulations were calculated to detect expansion (10,000 permutations and theta estimated from the data were used in the coalescent simulations). Lower *R*_2_ and rg values are expected for a population growth scenario (Harpending 1994; Ramos-Onsins and Rozas 2002). These analyses were performed in DnaSP 4.0 (Rozas et al. 2003).

## Results

### Polymorphism and haplotype diversity

A total of 279 *P. elegans* individuals from 14 European locations were sequenced. From this sample, 121 unique haplotypes of the COI gene fragment (600 bp) were identified. An expanded dataset included 20 additional individuals from three North American locations for a total of 299 sequences, 567 bp in length, with 123 unique haplotypes (GenBank accession numbers JN033571–JN033693). The most common haplotype, EUNA10, was shared by 36 European individuals and was found in all three Danish populations, Iceland, Sweden, and Plym Bay in the UK (English Channel). This haplotype was also observed in worms sampled from both populations on the East Coast of the United States (NAE). The second most common haplotype, EU11, was found in Denmark, Finland, France, the Netherlands, and the White Sea, Russia (35 individuals). Note that both EUNA10 and EU11 were found in populations differing in developmental mode (see Table 1). These two most common haplotypes also were found within the whole sample range in Europe and comprise 25% of all individuals sequenced.

We observed a large number of low frequency haplotypes within locations in Europe. Out of 121 haplotypes, 98 were detected only once in the European dataset (from only one individual of the 279 sequenced). Ninety percent of the haplotypes (109 out of 121) were found in only one population (11 of these were found from more than one individual). Populations from the North Sea, English Channel, and Wadden Sea had the highest percentage of population-specific low-frequency haplotypes. The Baltic Sea populations (Finland, Denmark, Germany, Sweden) shared many haplotypes (seven out of 12 shared haplotypes are found only in the Baltic Sea), and only one haplotype (EU8) was shared exclusively among the three populations in the UK and France. In most populations, haplotype diversity was high (Table 1). However, two European populations had low diversity with most individuals sharing the same haplotype. In Ängsö, Finland, 19 of 22 individuals sampled (86%) shared an identical haplotype (EU6) and in the sample of 20 individuals from Iceland, 13 shared haplotype EU1 and six shared haplotype EUNA10. Overall, populations with predominantly planktonic larvae had higher haplotype diversity than populations that also produced other larval types (*N*= 9, *U*= 0.000, *z*=–2.334, *P*= 0.020). However, nucleotide diversity was not significantly different (*N*= 9, *U*= 4.5, *z*=–1.167, *P*= 0.243). Haplotype diversity in the North American samples was somewhat lower than in most of the European samples (Table 1), but North American sample sizes were also relatively small and so estimates of diversity from these populations may not be reliable.

Mean sequence divergence (Tamura Nei model) within the total European dataset was 1.7%. Divergence between the European and North American haplotypes was noticeably higher: 5.3% (or 6.1% when excluding EUNA10, the haplotype that is shared with the European samples). Mean sequence divergence within the total North American dataset was 3.1%, higher than what we observed from the European sample.

### Haplotype network and phylogenetic analyses

**Figure 3:**
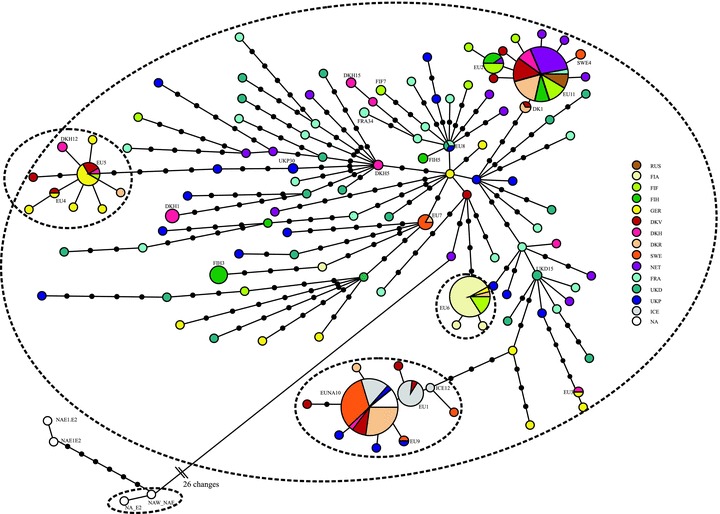
Haplotype network of the 123 COI haplotypes detected in *P. elegans*. Circle size is proportional to haplotype frequency and haplotypes with more than one individual are also named. Small black circles represent undetected intermediate haplotypes and lines connecting circles represent one mutational step unless otherwise specified. Circles are colored to represent sampling sites. Haplotypes found from more than one location are colored as pie charts with proportionally sized wedges representing the haplotype frequency in each population. Ovals with dashed outlines encircle clusters in the haplotype network which were also detected in phylogenetic analyses with strong (70% or greater) bootstrap support.

Figure 3 shows the minimum spanning haplotype network as calculated in Arlequin. The low sequence divergence among haplotypes is reflected in the network and haplotypes from different populations are intermingled. Arlequin detected many alternative connections among the European haplotypes due to the low level of divergence between them, but graphing all possible alternative connections would have made the network unreadable. The most common haplotype, EUNA10, was found from almost all populations and other linked haplotypes came from Iceland, the North Sea, the English Channel, and the Southern Baltic Sea, but not from Finland. The other common haplotype, EU11, is multiple mutational steps away from EUNA10. Moreover, the other North American haplotypes were not connected closely to EUNA10 and were clearly different from the European sequences. The European haplotype closest to the cluster of North American haplotypes is from the Netherlands (Fig. 3), and alternative connections (also 26 mutational steps to NAW_NAE) are from the UK (two haplotypes from UKP, one from UKD; not shown). Overall, the minimum spanning network included a large number of small nodes depicting the high frequency of singleton haplotypes noted earlier, and some medium frequency haplotypes (observed in two to eight individuals) that were detected in one population only. These singleton and low-frequency haplotypes are widespread throughout the network.

**Figure 4:**
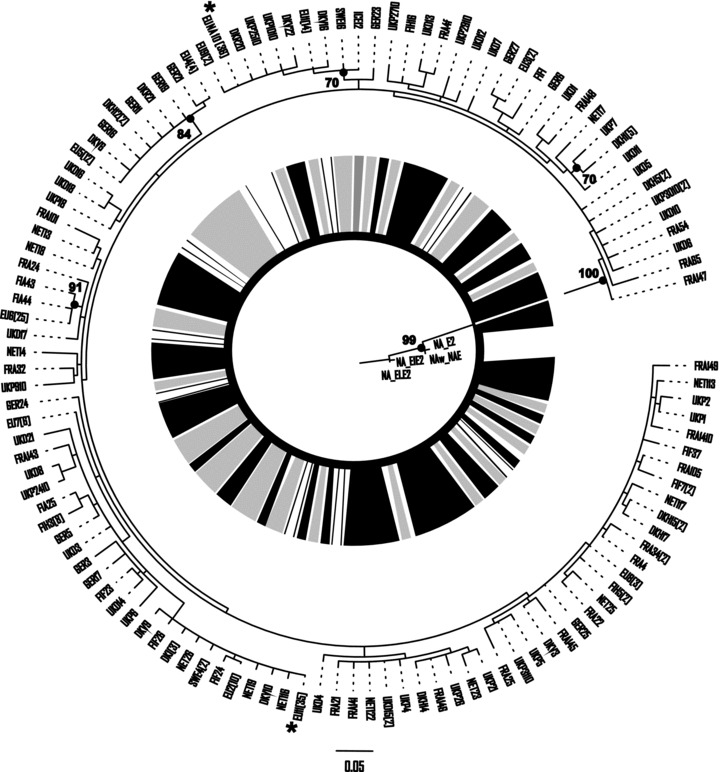
Maximum likelihood tree of COI haplotypes detected in *P. elegans*. Nodes marked with black dots indicate clades resolved with bootstrap values of 70% or higher. Dashed lines connect haplotype names to the branches and should not be interpreted as branch length. Numbers in the brackets following the haplotype name indicate the number of individuals observed with that haplotype. When no number is indicated, the haplotype was sampled only once. Asterisks indicate the two most common haplotypes: EUNA10 and EU11. Central color wheel indicates region of sampling: light gray = Baltic Sea (Northern + Southern); dark gray = Iceland; black = North Sea, English Channel and Wadden Sea; and white = shared European haplotypes. Note, the North American haplotypes in the circular phylogeny are in the center of the color wheel.

Phylogenetic analyses resulted in similar tree topologies regardless of which tree reconstruction method was used, therefore, only the results from the maximum likelihood analysis are discussed and shown (Fig. 4). As in the haplotype network, there was a clear separation between the European haplotypes and most North American haplotypes (other than EUNA10) and the European clade was well supported by bootstrap analysis (Fig. 4). Within the European clade, there was very little divergence and only a few groups were clearly resolved with high bootstrap support (Fig. 4, dots at supported nodes). The lack of bootstrap support at most nodes indicates limitations of the data for resolving relationships of the *P. elegans* haplotypes. However, this analysis also reveals clusters detected in the haplotype network. For example, one well-supported group contains almost all the Finnish Ängsö haplotypes (3 out of 4; EU6, FIA43, FIA44). Another well-supported group contains individuals from Germany and all of the three Danish populations, even though other haplotypes from these populations were also distributed elsewhere in the phylogenetic tree. In addition, most Swedish (except one) and all Icelandic haplotypes were included in a well-supported group, which also included EUNA10, one of the most common haplotypes also sampled from North America. The two most commonly encountered haplotypes (EUNA10 and EU11) did not group together (Fig. 4, asterisks).

### Population structure and demographic analyses

For the test of regional subdivision of sequence diversity in Europe (AMOVA), the populations were arranged into four groups according to geographical region (1. Northern Baltic Sea: Finland, 2. Southern Baltic Sea: Denmark, Germany, Sweden, 3. North Sea + Wadden Sea + English Channel: UK, France, the Netherlands, and 4. North Atlantic Ocean: Iceland, see Fig. 2). These results (Table 2) showed that most of the variation was found within populations (69.8%, *P* > 0.001) and that differentiation among the regions was significant although small (accounting for 8% of the molecular variance, *P*= 0.001). Additional significant variation among populations within each region (22.2%, *P* > 0.001) indicated that structure may also be present on smaller spatial scales.

**Table 2:** Analysis of molecular variation results. Four groups (regions) were used in the analysis: Northern Baltic Sea; Southern Baltic Sea; North Sea + Wadden Sea + English Channel; North Atlantic Ocean. Populations included in each region are indicated in Table 1.

Source of variation	Sum of squares	% of variance	Fixation index	*P*-value
Among regions	148.06	8.01	0.080	>0.001
Among populations in regions	230.19	22.21	0.241	>0.001
Within populations	864.15	69.78	0.302	>0.001

Analysis using the program BAPS detected seven genetic clusters in our data (with the probability of 0.99). Clusters were not based on sampling location, each cluster containing individuals from four to ten sampling locations. Two clusters were strictly Baltic, one containing most of the individuals sampled from the Finnish Ängsö site and the other containing most of the German samples, although German individuals were also placed into four other clusters (Table 3).

**Table 3:** Results from the BAPS genetic clustering analysis. The population code is underlined if 10 or more individuals from that population are in the cluster. If the code is in parentheses, only one to three individuals from that population are observed in the cluster.

	*N*	Population code	Northern Baltic	Southern Baltic	NS, WS, EC^1^	Atlantic Ocean	White Sea
Cluster 1	27	FIA, FIF (DKR, GER)	25	2			
Cluster 2	19	FIH (GER, UKD, UKP, FIA)	11	3	5		
Cluster 3	57	NET, FIF, DKV, DKR, FIH (DKH, RUS, SWE, UKD)	18	21	15		3
Cluster 4	43	UKD, FRA, DKH, UKP, NET (GER, FIH, FIF)	2	11	30		
Cluster 5	22	GER, DKV (DKH, DKR, FIF)	1	21			
Cluster 6	52	FRA, UKP, UKD, NET, DKH, SWE (GER, FIF, FIH, DKV)	5	14	33		
Cluster 7	59	ICE, SWE, DKR, UKP, DKV (DKH)		32	7	20	

1 NS = North Sea; WS = Wadden Sea; EC = the English Channel.

Demographic analyses suggested there has been recent population expansion in the populations from the UK and France. In these populations, Fu's *F*s values were negative and significant. Unimodal mismatch distribution curves for these populations also indicate that a recent population expansion may have occurred (see, e.g., Fig. S1). Lower *R*_2_ and rg values were also seen in these populations, but none of these values are significant. For these populations, Tajima's *D* and Fu and Li's neutrality tests were nonsignificant (Table 4), supporting an expansion hypothesis rather than possible selection. Most other populations had bi- or multimodal mismatch curves (characteristics of populations in demographic equilibrium) as well as nonsignificant neutrality test values (Table 4). The one exception is the Finnish Ängsö population which showed negative and significant neutrality test values (Tajima's *D*, Fu and Li). Truncated, left-skewed mismatch distribution curves were seen for the Finnish Ängsö and Iceland populations, indicating that most haplotypes were identical within these populations.

**Table 4:** Neutrality tests and mismatch distribution parameters. Coalescent simulations (10,000 permutations) were used to asses *P*-values for rg (Raggedness statistics) and *R*_2_.

Population	Fu's *F*s	Fu and Li's *F*	Tajima's *D*	rg	*R*_2_
FIA	0.093	−3.716*	−2.314**	0.369	0.171
FIF	0.777	−0.477	0.112	0.072	0.129
FIH	4.481	1.055	1.085	0.098	0.179
GER	−4.776	−1.509	−1.017	0.031	0.083
DKV	−0.696	0.539	0.959	0.027	0.166
DKH	0.387	−0.285	−0.141	0.078	0.125
DKR	4.452	−0.527	0.534	0.156	0.151
SWE	3.925	1.143	0.527	0.291	0.157
NET	−2.799	−1.975	−1.048	0.036	0.085
UKD	−11.426***	−1.048	−0.906	0.024	0.089
FRA	−14.231***	−1.486	−0.997	0.022	0.081
UKP	−7.748**	−1.038	−0.708	0.018	0.096
ICE	−0.060	−0.525	−0.090	0.201	0.155

* *P* < 0.02;

** *P* < 0.01;

*** *P* < 0.001.

## Discussion

Our analysis of partial mitochondrial COI sequences from *P. elegans* found little evidence for cryptic species. Sequence divergence was low particularly among European samples (1.7%), which originated from populations with different developmental modes. This modest degree of divergence is lower than within species divergence observed in similar studies of other polychaetes and of other poecilogonous species (discussed further below). In addition, the sequence divergence in European *P. elegans* was lower than threshold values used for delineating “potential” species in the DNA barcoding program (see Goetze 2003; Hebert et al. 2003; Costa et al. 2007; Carr et al. 2011).

However, average sequence divergence between samples from Europe and North America was approximately three-fold higher (5.3%). The higher divergence is not surprising given the geographic distance of the samples, but it may indicate a possible cryptic species in North America. Unfortunately, since our collections in North America were limited (three populations, each with six to seven sampled individuals), we are unable to make a strong conclusion about this result, and additional data from unsampled populations in North America and larger sample sizes are needed. However, the degree of divergence among the North American and European haplotypes provides perspective and strengthens our conclusion of poecilogony among European populations.

Several studies have investigated the level of sequence divergence in COI among closely related species of polychaetes. For example, between species divergence in the genus *Arenicola* is on the order of 14% (Luttikhuizen and Dekker 2010) and it is as high as 16% in *Pectoria koreni* (Jolly et al. 2005). Similarly, divergence between species in the *Eumida sanguinea* and *Marenzelleria* species complexes ranges from 6.5% to 18.5% (Nygren and Pleijel 2011) and 11.7% to 21.7% (Blank and Bastrop 2009), respectively. Carr and colleagues (2011) used a COI barcoding approach to survey broadly polychaetes collected from Canadian waters. On average, they detected 16.5% divergence between species, and within-species divergence ranged from 0% to 3.8%.

Divergence in COI sequence also has been used previously to address whether marine invertebrate taxa with observed developmental polymorphism are truly poecilogonous or actually cryptic species. For example, Schulze and colleagues (2000) found evidence for two distinct COI sequence clades among polychaetes in the genus *Streblospio* in North America. Sequence divergence between the two proposed species, a planktotrophic *S. gynobranchiata* and poecilogonous *S. benedicti* was approximately 20% and within-species divergence was ∼5%. Both planktotrophic (planktonic) and lecithotrophic (brooded) larvae have been documented within the *S. benedicti* clade. COI sequence data have also been employed to investigate the potential for cryptic species in the marine gastropod genus *Alderia*. Ellingson and Krug (2006) found evidence for two well-supported species-specific sequence clades for *A. willowi* and *A. modesta*, which were 20.6% divergent from one another. Within each clade, sequence divergence was less than 5%. Although the *A. modesta* clade produces only planktonic larvae, slugs in the *A. willowi* clade produce multiple larval types. In this case, both sequence data and morphological evidence (Krug et al. 2007) indicated that *A. willowi* is poecilogonous.

The distribution of genetic variation among *P. elegans* populations provides a second line of support to a conclusion that this species is poecilogonous. We found high haplotype diversity in *P. elegans* in Europe although nucleotide diversity was low. This pattern was consistent with the observed high proportion (90%) of low-frequency haplotypes found in one or a few populations. On the other hand, two haplotypes dominated the sample (25% of individuals). Both of these haplotypes had a broad distribution in many of the European populations we sampled and EUNA10 was also sampled from North America. We also found four haplotypes that were shared among populations characterized by different modes of development, including the most common haplotypes, EUNA10 and EU11. Similar findings were reported for the poecilogonous *A. willowi*, for which shared haplotypes were observed by Ellingson and Krug (2006) between slugs producing different larval types; and also for the polychaete *B. proboscidea*, where there was no association between sequence clades and developmental mode (Gibson et al. 1999; Oyarzun et al. 2011). In our study, we have compared patterns of genetic differentiation among populations which we have characterized by the predominant developmental mode. Ideally, we would have both sequence and developmental mode information for all individuals in our study. Nevertheless, the low levels of sequence divergence and the fact that haplotypes are shared among populations differing in developmental mode and throughout the broad sampling area (Baltic Sea, Wadden Sea, North Sea, and Atlantic Ocean) are inconsistent with a hypothesis of cryptic speciation. Instead, our data support the hypothesis that *P. elegans* populations, particularly those in Europe, are poecilogonous.

However, the significant AMOVA results among regions and among populations within regions indicate that there is genetic structuring at multiple spatial scales within Europe. Our sampled populations cover both a latitudinal and longitudinal gradient and experience different environmental conditions, such as differing temperature extremes, salinities, and substrata, so potential barriers to dispersal may be present between our sampling localities. The AMOVA indicated significant Φ_ST_ among sea regions but it only accounted for 8% of variation in the model. The BAPS-based analysis also suggested the presence of multiple genetic clusters within Europe. On the whole, the clusters did not correspond to our definition of populations by collection locality. The only exceptions were for the haplotypes obtained from Ängsö (Finland), Sweden, and Iceland populations, which were also found in some of the few well-supported clades in our maximum likelihood phylogeny. Overall, these results indicated that the bulk of genetic variation in *P. elegans* resided within populations.

We observed especially low genetic diversity among the worms in the two marginal populations: Ängsö, being located in the inner parts of the Finnish Archipelago, and Iceland, further away from the bulk of the sampled localities in the Atlantic Ocean. Mismatch distribution curves for the sequences from these populations were strongly skewed to the left showing the reduced diversity, which likely reflects limited gene flow to these populations because of their marginal distribution or a recent (re)colonization or other bottleneck-like event. The significant neutrality test values seen in the Ängsö population indicate that selection could also cause this pattern. However, if selection were the cause, we would expect the other two nearby Finnish populations (the distance from FIA to FIF is only 20 km) to show similar results. Alternatively, asexual reproduction could lead to lower diversity. Asexual reproduction has previously been observed in some *P. elegans* populations (e.g., Rasmussen 1953; Hobson and Green 1968; Anger 1984; Lindsay et al. 2007). Wilson (1983) reported that asexual reproduction predominates in False Bay, Washington (west coast United States). In our study, only one *P. elegans* COI haplotype was found from False Bay, but the sample size was low (*N*= 7) and some possible variation may have gone undetected, so it is impossible to say at present whether an increased frequency of asexual reproduction leads to a greater reduction in genetic diversity.

Several studies have compared genetic diversity among marine invertebrate species with different developmental modes and generally found higher genetic diversity in planktonic-developing species compared to brooded or direct-developing species (Hellberg 1996; Hoskin 1997; Arndt and Smith 1998; Ayre and Hughes 2000; Kyle and Boulding 2000; Collin 2001; Breton et al. 2003; Ellingson and Krug 2006; Lee and Boulding 2009). For example, among snails in the genus *Littorina*, Boulding and colleagues (Kyle and Boulding 2000; Lee and Boulding 2009) observed that genetic diversity is higher in the planktotrophic species *L. scutulata* and *L. plena* than in the direct-developing species *L. sitkana* and *L. subrotundata*. Lee and Boulding (2009) suggested that effective population size (*N_e_*) is larger in planktonic-developing species than in direct-developing species, and as a result, the effects of genetic drift are diminished and a larger number of rare haplotypes are more likely to be retained. In another notable example, Ellingson and Krug (2006) found genetic diversity to be higher in the fully planktotrophic *A. modesta* compared to genetic diversity in the poecilogonous *A. willowi*.

Our analysis with *P. elegans* suggests that the patterns of genetic diversity and development mode may be correlated within a poecilogonous species as well. Haplotype and nucleotide diversity were lowest in Ängsö, Finland population for which brooding is the predominant mode of development, although reduced diversity in this population could also be the result of other demographic factors (see above). In contrast, populations showing a predominantly planktonic developmental mode were more diverse than the Ängsö population as well as those populations showing multiple developmental modes. Fu's *F*s test gave significant negative values for the three populations with predominantly planktonic larvae (UKD, FRA, UKP), indicating an excess of haplotypes, while sequences from these same populations had unimodal mismatch distributions with large numbers of pairwise differences. A pattern of significant Fu's *F*s and nonsignificant Fu and Li's test suggests that the populations may have undergone recent expansion and have high *N_e_* and that background selection is not likely (Rogers and Harpending 1992; Fu 1997). The populations with multiple developmental modes typically had bimodal mismatch distributions with both highly similar and highly differentiated haplotypes.

Although we cannot be certain that our definition of predominant developmental mode for the different populations is correct, our results are interesting because they reflect the general expectations of how developmental mode can influence the genetic diversity within populations, as well as population genetic structure and gene flow among populations of a species (see reviews by Avise et al. 1987; Bohonak 1999; Pechenik 1999). For example, many empirical studies have shown that gene flow is greater for species with pelagic larvae resulting in less genetic structure among populations when compared to species with brooded larvae (e.g., Hellberg 1996; Hoskin 1997; Arndt and Smith 1998; Ayre and Hughes 2000; Collin 2001; Ellingson and Krug 2006). However, the generality of this expectation depends upon dispersal during the pelagic phase. When dispersal is not realized during the pelagic phase, exceptions can occur, and studies highlighting such contrary findings are not uncommon (e.g., Porter et al. 2002; Miller and Ayre 2008; Weersing and Toonen 2009; and see Hellberg et al. 2002 for review).

Our analysis suffers from the lack of individual level data for developmental mode and has only tentatively defined populations as predominantly planktonic, brooding, or both. We cannot rule out the possibility that other developmental modes are predominant at other times of the year or that developmental mode might fluctuate temporally either due to phenotypic plasticity or population turnover. In this regard, it is interesting to note that Morgan and colleagues (1999) found only brooded larvae in the Plym Bay population during repeated sampling in 1997, while in 2010, we observed only planktonic larvae in the same population. In our analyses, the Plym Bay population had high haplotype diversity similar to that seen in other planktonic populations. The change in developmental mode noted in this population may be explained by the ability of *P. elegans* to re-colonize rapidly disturbed areas (Desprez et al. 1992; Kube and Powilleit 1997). For other populations that have been repeatedly sampled, we found no evidence of a change in developmental mode. We observed only planktonic larvae at the Drum Sands and Somme Bay populations, the same mode reported by Bolam (2004) and Morgan et al. (1999), respectively. Like Rasmussen (1973), we found that Danish populations had both planktonic and brooded larvae, as well as intermediate larvae. *Pygospio elegans* from Gullmar Fjord, Sweden, have been previously reported to have intermediate and brooded larvae (Hannerz 1956), but we did not observe reproductive females or larvae during our collection.

Our study is not the first to consider whether *P. elegans* is poecilogonous. Morgan and colleagues (1999) studied four *P. elegans* populations from the English Channel. They found that the Plym Bay and Ryde Sand populations from UK were strictly brooding while the French Somme Bay population and English Swale Bay populations produced only planktonic larvae. Based on allozyme analysis, they observed significant population structure but found no evidence of cryptic species in the English Channel. In our study, we widened the sample area in an attempt to increase the chance of finding cryptic species if such existed in Europe, but our conclusions uphold the previous results even at the broader spatial scale. Although the previous experimental study by Anger (1984) led her to suggest cryptic species, (since changes in temperature or salinity did not induce changes in developmental mode), we found little evidence to support her view. We feel that alternative explanations for her results should be considered: for example, developmental mode may not be a plastic trait, or if it is, temperature and salinity may not be the appropriate cues to trigger a plastic response.

## Conclusion

In this study, we analyzed DNA sequence variation from *P. elegans* sampled from a broad geographic range. Very little sequence divergence was observed among individuals despite variation in developmental mode observed both among and within populations. Using a DNA barcoding criterion based on sequence divergence, there is no evidence for cryptic species in this taxon. In addition, haplotype network and phylogenetic analyses did not point to potentially cryptic species as no clear clustering of haplotypes was resolved among the European samples. Divergence of North American *P. elegans* may warrant further study. Given these results, we conclude that developmental polymorphism in *P. elegans* is likely a true case of poecilogony. These results also confirm the previous population genetic study of Morgan and colleagues (1999) which used genetic methods with lower resolution and had a more restricted geographical scope.

## Supporting Information - Evolutionary Analysis

**Figure S1.** Examples of mismatch distribution curves (analyses for growth-decline scenario): observed pairwise nucleotide site differences and the expected curves for growing or declining population (Rogers and Harpending 1992).
