# Supplementary material for: Phylogenetic analysis of cryptic speciation in the polychaete Pygospio elegans
Source: Ecol Evol. 2012 May;2(5):994–1007. doi: 10.1002/ece3.226 (PMC3399165; doi:10.1002/ece3.226)
Supplement: Supplementary file 1 [file ece30002-0994-SD1.doc]

Supplement 1. Examples of mismatch distribution curves (analyses for growth-decline scenario): observed pairwise nucleotide site differences and the expected curves for growing or declining population (Rogers and Harpending 1992).


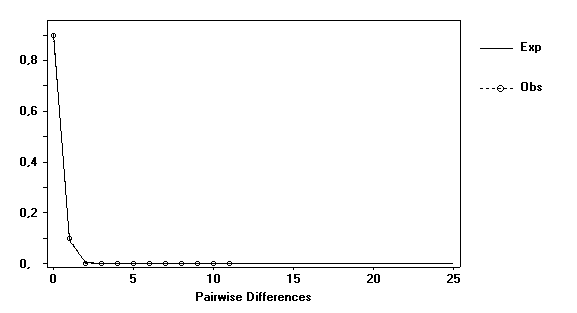


1. Iceland (ICE)


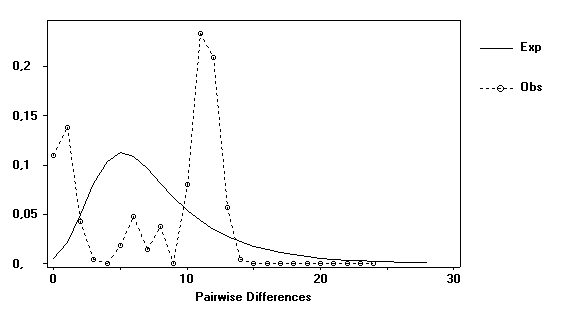


1. Fårö, Finland (FIF)


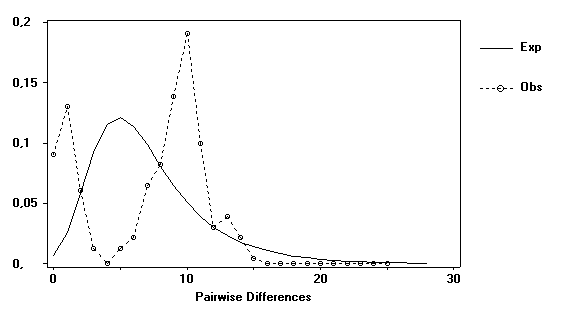


1. Germany (GER)


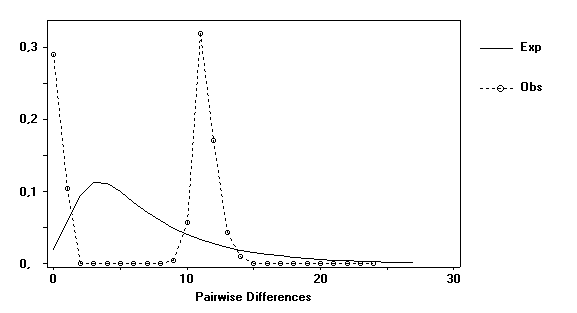


1. Rorvig, Denmark (DKR)


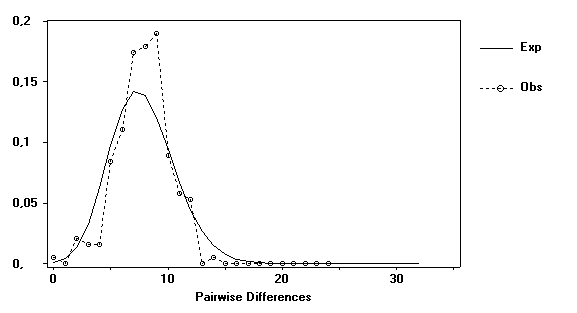


1. Drum sands, UK (UKD)


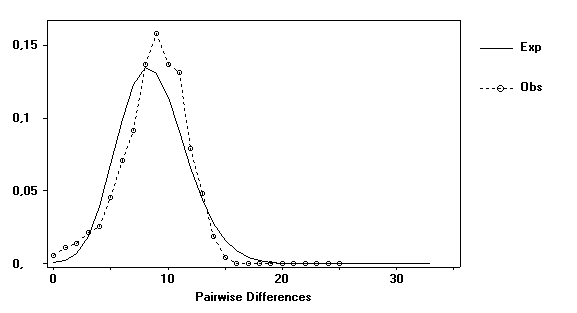


1. UKD+UKP+FRA data combined
